# Schematic eye-gaze cues influence infants’ object encoding dependent on their contrast polarity

**DOI:** 10.1038/s41598-017-07445-9

**Published:** 2017-08-04

**Authors:** Christine Michel, Sabina Pauen, Stefanie Hoehl

**Affiliations:** 1 Institute of Psychology, Heidelberg University, Hauptstrasse 47–51, 69117 Heidelberg, Germany; 2 Max Planck Institute for Human Cognitive and Brain Sciences, Stephanstraße 1a, 04103 Leipzig, Germany

## Abstract

We examined infants’ sensitivity to eye-gaze direction and its influence on object processing in 4-month-old infants by manipulating low-level properties of gaze cues. Infants were presented with two kinds of stimuli that either did or did not cue novel objects. The movement of a schematic image of two eyes (two black circles each moving on a white oval background) led to an enhanced processing of the cued object. A cue with reversed polarity (two white circles each moving on a black oval background) elicited distinctly weaker effects. Results highlight infants’ specific sensitivity to isolated eye gaze which furthermore facilitates object encoding. It is suggested that this sensitivity relies on the typical perceptual pattern of eyes, the black pupil on a white sclera.

## Introduction

Human beings are remarkably sensitive to eye-gaze direction of another person right after birth^1, 2^. This ability is crucial for infants’ ability to interact with and to learn from others: On the one hand, detecting that the gaze of another person is directed toward the self signals the infant that s/he is the addressee of a communication. This may make the infant highly receptive for information provided by the interaction partner^3, 4^. On the other hand, perceiving averted gaze can guide infants’ attention toward objects in the environment and facilitate the encoding of such socially cued objects^5, 6^. Here, we further investigate infants’ sensitivity to eye-gaze direction and its influence on the processing of novel objects. We specifically examine the role of the particular contrast of eyes, a black pupil on a white sclera, for object encoding in 4-month-olds.

As part of his proposed Mindreading System, Baron-Cohen suggested that infants posses a neural module dedicated to detecting eyes or eye-like stimuli from birth^1, 7^. This Eye Direction Detector (EDD) is sensitive to the specific contrast of the dark region of the pupil and the light region of the sclera and encodes their relative spatial position. The module was considered a fundamental building block for later developing social cognitive abilities like shared attention (SAM: Shared Attention Mechanism) or Theory of Mind (ToMM: Theory of Mind Mechanism)^7^.

Later accounts by other authors, however, discarded this modular view. For instance, Farroni and colleagues showed in a series of experiments that infants’ gaze processing depends on the eyes being situated in an upright face and does not function in the context of an inverted face^8–11^. These results are in line with studies in adults showing that gaze cueing effects are reduced when faces are inverted^12–14^. It was concluded that gaze perception may be an integrated part of a more general communication system, rather than relying on a specific module on its own in infancy^2, 10, 11^. Based on this assumption, it was suggested that infants can use eye gaze as a communicative cue only in the context of an upright face^3^.

But even though inverted faces seem to disrupt infants’ gaze processing, this does not show that an intact, upright face is actually required for infants to respond to gaze cues. It is possible that face inversion impedes the use of gaze cues in infancy, but that eyes without the context of the face still activate a neural system dedicated to gaze processing. Behavioral evidence for this assumption comes from Kingstone, *et al*.^12^. Schematic eyes without the context of a face elicited gaze cueing effects in split-brain patients. A recent study by Fawcett, *et al*.^15^ provided data of 6- and 9-month-olds showing a specific pupillary response to schematic eyes but not to perceptually similar squares. On the neurophysiological level, neurons responding specifically to the direction of isolated eyes as well as gaze direction in the context of a face were found in the superior temporal sulcus of the macaque monkey using single cell recordings^16, 17^. This discovery lead to the idea of a Direction of Attention Detector (DAD)^18^. The suggested function of the DAD is to extract the focus of attention of another person through hierarchically combining information from eye gaze, head and body direction. Thereby, information from eye gaze overrides input from other cues via inhibitory connections^17, 18^. Compared to the EDD proposed by Baron-Cohen^7^ that focusses on the eye-gaze direction, the DAD can be seen as a more general mechanism which takes into account information from several different sources (eye-gaze direction, head and body posture)^18^.

Some preliminary evidence for gaze sensitive neural systems as purported in the DAD in human infants comes from studies on infants’ object processing depending on cues of social attention. In a study by Wahl, *et al*.^19^, 4-month-old infants saw a person shifting her head and eye gaze toward or away from a novel object. These objects were presented again for a second time and responses to the previously cued and not-cued objects were analyzed, using both event-related potentials and looking times as dependent measures. An enhanced Negative Central (NC) component was found for previously not-cued objects. The NC component is supposed to reflect attentional processes^20, 21^. Hence, processing of the previously not-cued object required the involvement of additional neural resources. The cued object, in contrast, was already processed more effectively while the person was looking at it. On the behavioral level, infants looked longer to the previously not-cued than to the previously cued object. Consistent with the neural results, the not-cued objects were not as effectively processed as the cued ones. When presented for a second time, these objects therefore appeared more novel and, thus, more interesting to the infants, as indicated by longer looking times.

The simultaneous shift of eye gaze and head orientation facilitated the encoding of the cued objects. Intriguingly, when using the same paradigm but presenting *either* gaze shifts *or* head turn as the social cue in 4-month-olds, identical results were obtained. Again, the previously not-cued object elicited longer looking times^22, 23^ or enhanced neural activity^22, 24^. This suggests that both cues, head orientation and eye-gaze direction, independently affect infants’ object encoding in a similar way. These findings are in favor of the existence of neural systems that encode eye gaze and head orientation as proposed in the DAD. The inhibitory connections between those neurons may just not yet be developed^22^. However, as eyes were always presented in the context of an upright face, these results leave open whether infants’ sensitivity to gaze cues and its impact on object encoding relies on neural systems that specifically encode gaze direction as proposed by the EDD or the DAD^7, 18^ or if a more general mechanism is activated that requires the context of a face to respond to gaze cues^2, 3, 10, 11^.

In our study, we aim to test if infants possess a specific sensitivity to eye-gaze direction even without the context of a face. Such a sensitivity may help infants to detect the focus of attention of another person and relate it to an object in the environment. The eye-gaze direction of another person will therefore cue infants’ attention to this object which will facilitate the processing of this object^5, 6^. We applied a well-established paradigm to investigate the influence of social cues on object processing^19, 22, 24^. However, instead of complete faces we presented schematic eyes, two black circles moving on a white oval background, as the cue (natural-contrast condition)^12^. In a second condition, we reversed the typical black and white contrast and presented two white circles moving on a black oval background (reversed-contrast condition). The specific black and white contrast is characteristic for human eyes^25, 26^ and inverting the contrast is known to alter infants’ behavioral and neural responses to faces^9, 27, 28^, emotional eyes with direct gaze as well as the distinction between direct and averted gaze^29^. In addition, inverting the contrast polarity of eyes led to reversed cueing effects in adults^30^. In our study, we examine the influence of eye gaze polarity on infants’ attention direction and encoding of novel objects.

If infants at 4 months of age already possess a specific sensitivity to eye-gaze direction whose function is to promote object encoding, we expect schematic eyes to affect infants’ processing of novel objects even without the context of a face. In line with previous research^22^, this would be reflected in longer looking times to objects which were previously not cued by schematic eyes when presented for a second time. If furthermore this sensitivity is driven by the low-level feature of the particular contrast of a black pupil on a white sclera, reversing this contrast should disrupt the influence of the cue on infants’ object processing. The reversed cue may still guide infants’ attention as infants’ attention can be biased by low-level features like the perceptual weight of a visual stimulus^31^ or movement^10^. Results in the reversed-contrast condition will therefore inform us about the influence of perceptual weight and movement on object encoding in the absence of eyes.

## Methods

### Participants

The final sample consisted of 42 infants (20 female). The *natural-contrast condition* and the *reversed-contrast condition* were tested between subjects with 21 infants included in each condition. Sample size was based on past research using the same paradigm^24^. All infants were born full-term (>37 weeks). The average age was 4 months and 12 days with an age range of 4 months and 0–31 days. Another 9 infants had to be excluded from the final sample due to failure to calibrate the infant (n = 5) or because they did not provide sufficient analyzable data (n = 4). Written informed consent was collected of all participants’ parents. The experiment was approved by the ethics committee of the Fakultät für Verhaltens- und Empirische Kulturwissenschaften, Heidelberg. All methods were performed in accordance with the relevant guidelines and regulations.

### Apparatus, procedure and stimuli

To test the influence of schematic eyes on infants’ attention and object processing, we employed a paired-preference paradigm to measure looking times to objects that were previously either cued or not-cued by a central stimulus^19, 22, 24^. Therefore, a central cue (i.e. a pair of schematic eyes) was presented simultaneously with two novel objects, one object to each side. The central cue moved to one side (i.e. the circles moved to one side of the oval background), thereby cueing one of the objects and moving away from the other one. Both objects were presented again and relative looking times to the previously cued and not-cued object were measured. Stimulus presentation, timing and analyses of data were adopted from Hoehl, *et al*.^22^ and Wahl, *et al*.^19^.

During the experiment, infants sat on their caregiver’s lap in a dimly lit room in approximately 60 cm distance of a Tobii T60 eye tracker. The eye tracker recorded data with a 60 Hz frequency. Stimuli were presented using Tobii Studio 2.2.8 (Tobii Technology AB, Danderyd, Sweden) on a 17” TFT screen.

**Figure 1:**
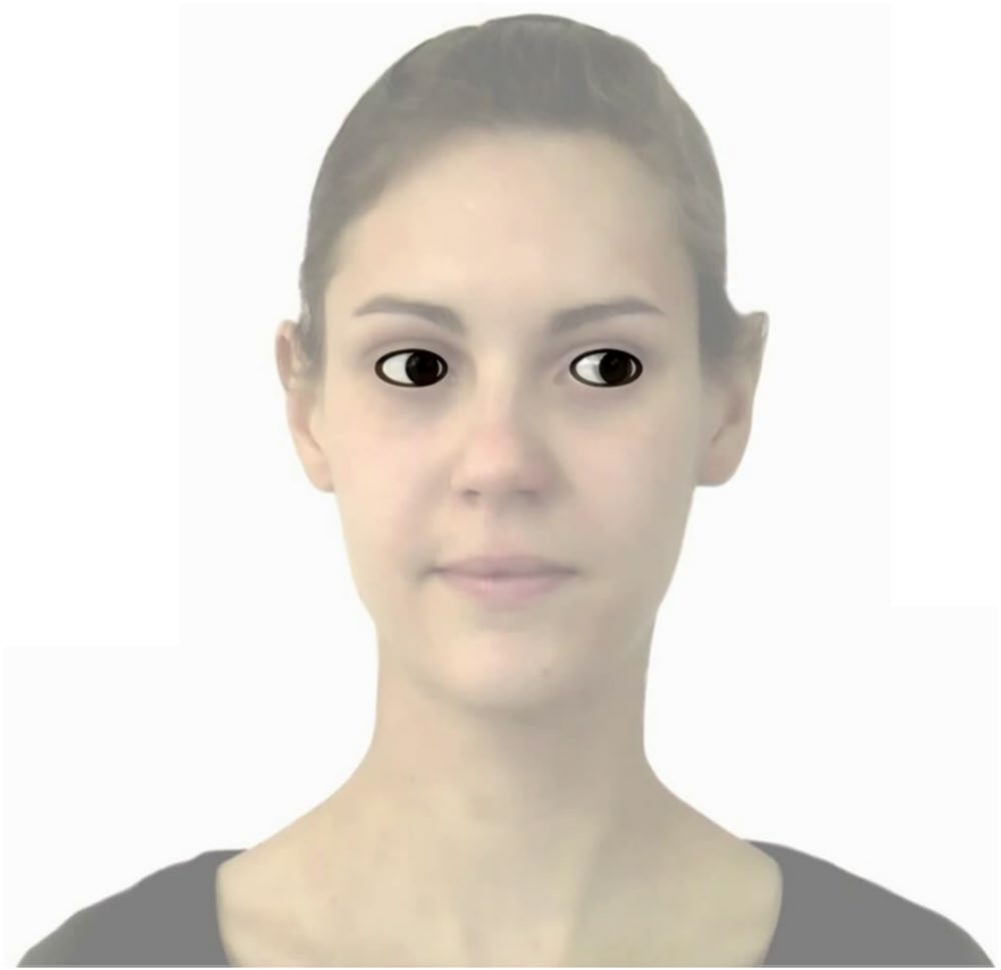
Overlay of the schematic eyes used in the current study in the natural-contrast condition and the stimulus used in the eye gaze condition by Hoehl, *et al*.^22^. The size and the movement of the schematic stimuli in the current study match the ones of the eyes in the video of the real person.

Infants were calibrated with a 5-point-calibration using a pattern of dots and a sound as the calibration stimulus. Each of the 8 trials started with a static black star (5.6° × 5.6°) on a white background presented in the middle of the screen for 2 seconds accompanied by an attention-grabbing sound. In the *natural-contrast condition*, the central cue consisted of two black transparent ellipses on a white oval background that marked the borders of each eye. Each ellipse contained a black circle in their middle. The entire shape appeared like eyes. The size, the position and the movement of the eyes were identical to those of the eyes of the person presented in Hoehl, *et al*.^22^ (Fig. 1). Each eye covered 1.8 cm × 0.9 cm (1.7° × 0.9°) of the screen. In each trial, two novel objects were presented, one to each side of the eyes. The objects were of a maximal width of 6.03 cm (5.8°) and a maximal height of 7.63 cm (7.3°). Previously cued and not-cued objects did not differ in the area they covered, *t*(14) = −0.858, *p* > 0.250.

**Figure 2:**
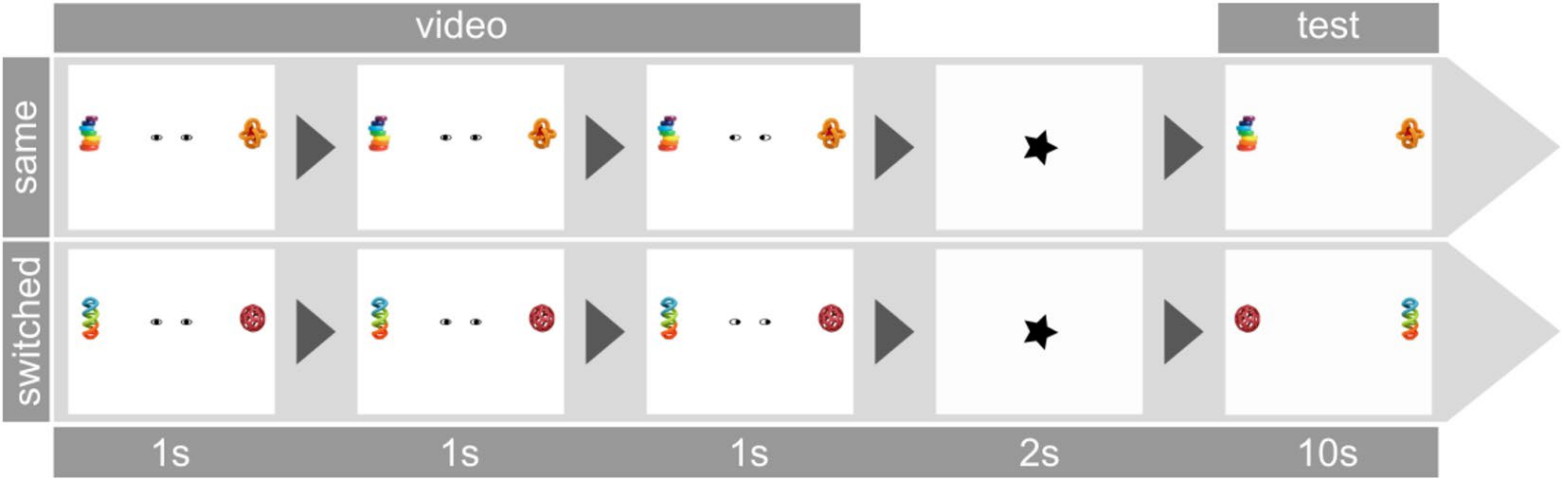
Examples of a trial in the natural-contrast condition with objects staying in the same location or switching positions in test. Stimulus presentation in the reversed-contrast condition was identical except for the contrast polarity of the central cue.

**Figure 3:**
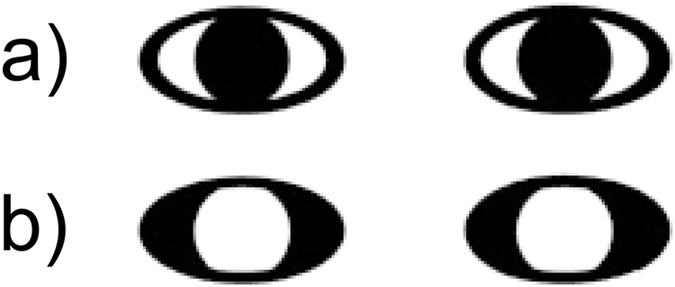
Central cue in the natural-contrast condition (**a**) and the reversed-contrast condition (**b**).

In the cueing phase of the trial, the circles were located in the middle of the ellipses appearing to look at the infant for 1000 ms (see Fig. 2 for a scheme of the trials). During the next 1000 ms the circles moved with constant speed to one side. As the circles reached the end of the ellipses, they partly disappeared behind the borders of the ellipses, such as pupils disappear when looking to the side. The circles thereby cued one object and moved away from the other one. This end frame was presented for another 1000 ms. Afterwards, a rotating star appeared for 2 seconds to focus infants’ attention back to the center of the screen. In the subsequent test phase, both objects were presented again simultaneously for 10 seconds. In half of the trials, the position of the objects was switched between cueing and test phase to rule out side effects. Trials were counterbalanced in such a way that not more than two times in a row did the circles move to the same side, did objects switch positions in test or did the previously cued object appear on the left/right side in test. The *reversed-contrast condition* differed only regarding the contrast of the central cue (Fig. 3) as white circles moved on a black oval background. In both studies, the cued object was defined as the one in the direction of the movement.

To control if infants have any preference for specific test objects *per se*, we presented the pairs of objects used in the study to an independent sample of 17 4-month-old infants (9 female, mean age 4 month 14 days, age range 4 months 0 days – 4 months 29 days). Data analysis was done in exactly the same way as for the cueing experiment. No main effects or interactions were found, all *p*s > 0.250. This ensures that any differences in looking times found in the cueing experiment are in fact induced by the central cues.

### Data Analysis

We measured relative fixation times to the previously cued and the previously not-cued object during test. Identical Areas of Interest (AoIs) were employed for all objects. AoIs were defined 1° visual angle larger than the maximal dimensions of the objects (6.8° width × 8.3° height). Fixations were created using a Tobii filter with a velocity and distance threshold of 35 pixels^19^ which also interpolated missing data for data segments below 100 ms.

A trial was included in the final analyses if infants fixated the screen for a minimum of 200 ms during each part of the cueing phase (eyes to front, movement of eyes, end frame with eyes to the side) and to at least one object during test. On average, infants contributed six trials to the final analyses. In the natural-contrast condition, 14 infants provided 6 or more analyzable trials. In the reversed-contrast condition, 17 infants provided 6 or more analyzable trials.

A participant was included in the final sample if s/he provided data for at least one trial with objects staying in their positions and to at least one trial with objects switching their positions from cueing to test phase. We used the software R^32^ to extract relative looking times.

To investigate differences in looking times to the objects in the paired-preference test, relative looking times (cumulative fixation length within the AoI during test relative to the overall fixation length to the screen in this trial during test) were used as the dependent variable. This way, the overall amount of time infants spent looking on the screen was taken into account^19, 22^.

### Data Availability

All data generated or analyzed during this study are included in its Supplementary Information files.

## Results

We conducted a mixed ANOVA with the within-subject factors cue (cued vs. not-cued object) and position (same vs. switched) and the between-subject factor contrast condition (natural vs. reversed) on relative looking times to the objects in test. Level of significance was set at *p* < 0.05. Means and standard errors of each factor level for each condition are depicted in Table 1. P-values were Bonferroni-corrected for post-hoc contrasts.

**Table 1:** Average relative looking times (standard errors) to objects in each combination of cue (cued and not-cued) and object position (same and switched) in both contrast conditions (natural and reversed).

Contrast condition	Cued		Not-cued	
	Same	Switched	Same	Switched
natural contrast	0.36 (0.03)	0.33 (0.03)	0.46 (0.04)	0.52 (0.03)
reversed contrast	0.30 (0.03)	0.44 (0.04)	0.51 (0.03)	0.39 (0.03)

The interaction of position x cue x contrast condition reached significance, *F*(1, 40) = 7.23, *p* = 0.010, ƞ² = 0.15. To dissolve this interaction, we conducted repeated-measures ANOVAs for each contrast condition with the within-subject factors cue (cued vs. not-cued object) and position (same vs. switched).

### Natural-contrast condition

**Figure 4:**
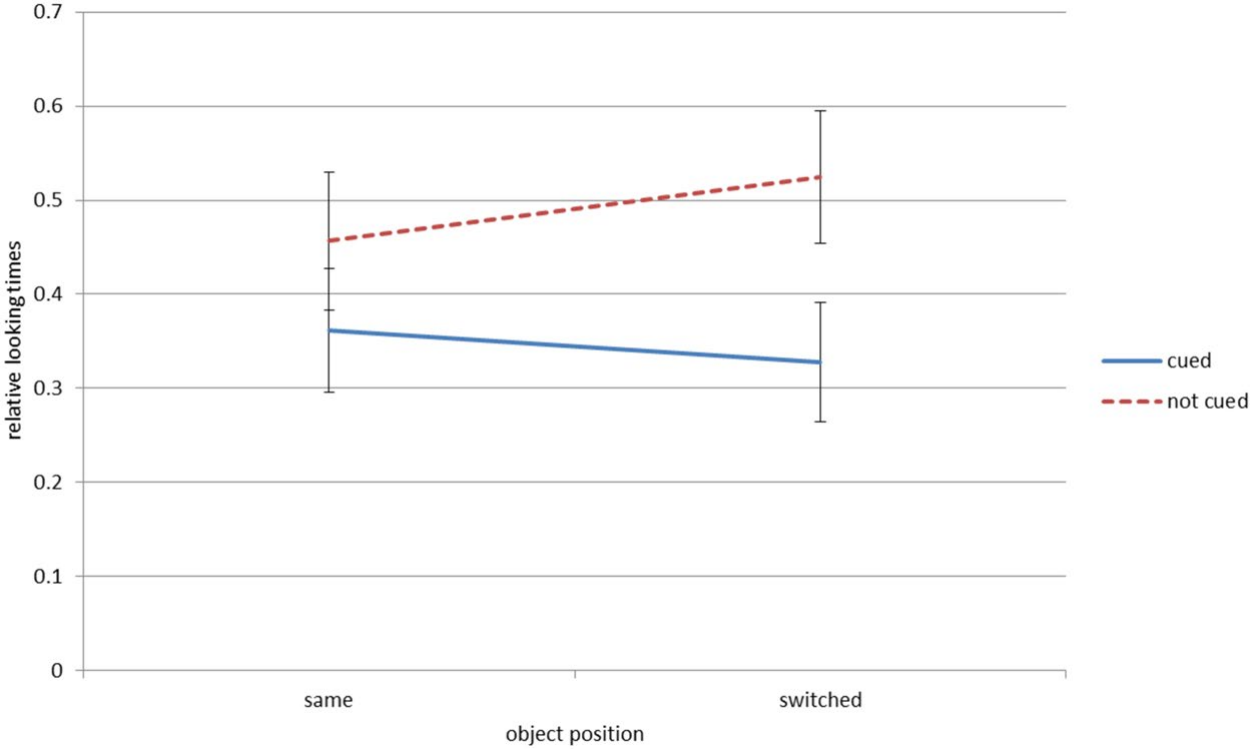
Natural-contrast condition: graphs of the mean relative looking times for the previously cued and not-cued objects in trials with objects staying at the same position or switching positions. Error bars represent 95% confidence intervals.

In the natural-contrast condition, the main effects of position, *F*(1, 20) = 4.65, *p* = 0.043, ƞ² = 0.19, and of cue, *F*(1, 20) = 12.39, *p* = 0.002, ƞ² = 0.38, were significant with longer looking times for not-cued objects compared to cued objects. Furthermore, looking times were longer in trials where objects switched positions at test compared to trials with same object positions during cueing phase and test. The interaction of position x cue did not reach significance, *p* > 0.250 (See Fig. 4).

### Reversed-contrast condition

**Figure 5:**
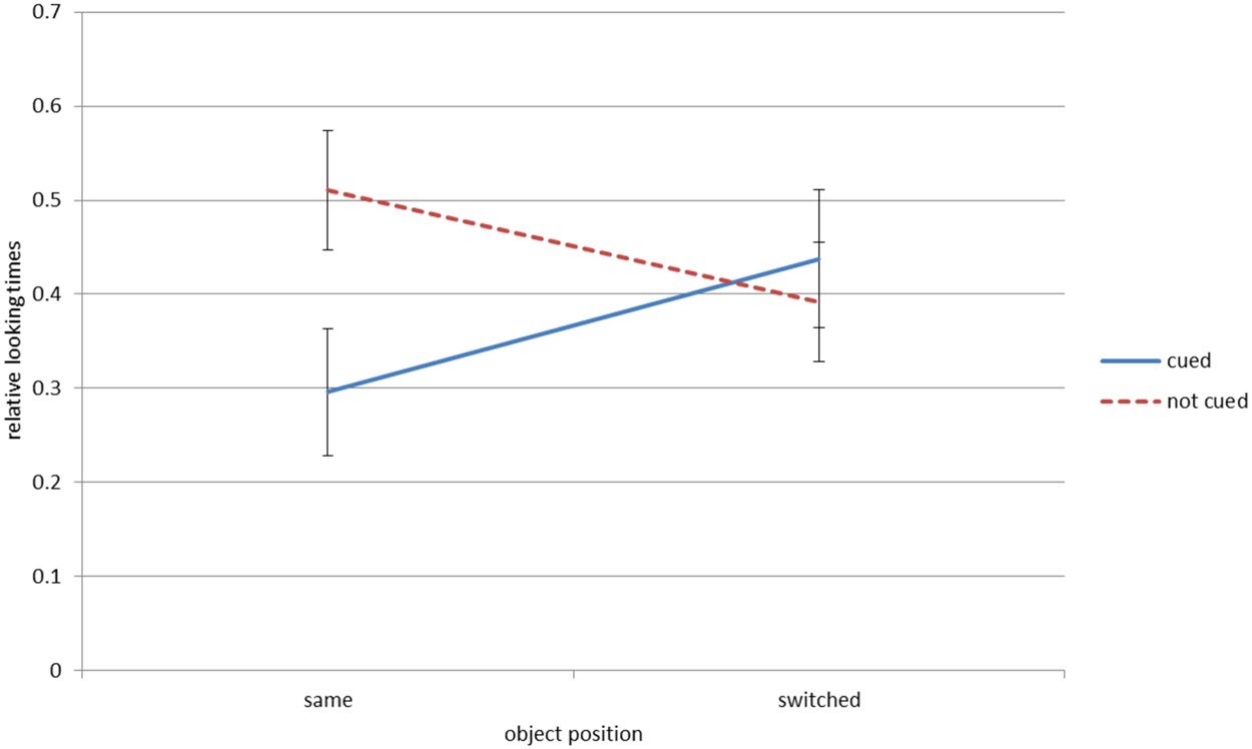
Reversed-contrast condition: graphs of the mean relative looking times for the previously cued and not-cued objects in trials with objects staying at the same position or switching positions. Error bars represent 95% confidence intervals.

We neither found a significant main effect of position, *p* > 0.250, nor cue, *p* = 0.057. However, the interaction position x cue reached significance, *F*(1, 20) = 7.88, *p* = 0.011, ƞ² = 0.28. Infants looked longer to previously not-cued objects than to previously cued ones when the objects stayed at the same position at test, *t*(20) = −3.53, *p* = 0.004, Cohens *d* = 0.77. No such difference was found in trials with objects switching positions, *t*(20) = 0.72, *p* > 0.250 (see Fig. 5).

## Discussion

We investigated the influence of gaze direction of schematic eyes with a natural contrast (black circles moving on a white oval background) and a reversed contrast (white circles moving on a black oval background) on infants’ attention and object processing. It was only in the natural-contrast condition that infants clearly showed facilitated object processing due to the dynamic gaze shift.

In the natural-contrast condition we found a significant main effect of cue with longer looking times for previously not-cued compared to previously cued objects. In line with the existing literature^19, 22, 24^, longer looking times to previously not-cued objects may reflect infants’ increased interest in these objects as they appeared more novel to them than the previously cued objects. Importantly, this main effect was not qualified by an interaction with object position, indicating that infants processed cued and not-cued objects differently irrespective of their location. Thus, infants encoded the objects and not their position. The finding that infants generally looked longer at objects with switched compared to same locations at test suggests that infants noticed the changed positions leading to higher overall scanning of the objects.

Even though both conditions only differed with regard to contrast-polarity of the central cue, different results were obtained in the reversed-contrast condition. As no main effect of cue was found, results do not fully support an influence of the cue on infants’ encoding of the objects like it was found in the natural contrast condition. Instead, the interaction between the position of the objects and the cueing information reached significance. In trials with unchanged object positions during test infants looked longer to the object in the previously not-cued direction (i.e. to the direction of the black part of the cueing stimulus). In trials with changed object positions, no such effect was found. This result shows that schematic eyes with reversed contrast did affect infants’ attention, but object encoding was not influenced by the reversed contrast cue as it was by the natural contrast cue. There are two possible factors which may have elicited infants’ preference for the previously not-cued object in same position trials. First, infants’ attention may have been guided by the perceptual weight of the stimulus. As Jakobsen, *et al*.^31^ showed, young infants’ attention is directed to the perceptually heavier side of a stimulus. The perceptually heavier part of the stimulus in the reversed contrast condition was the black part of the sclera, i. e. the side opposite to the movement direction. Infants’ attention may therefore have been shifted to the not-cued side, resulting in longer looking times to this side during test. This explanation is in line with reversed cueing effects in adults when contrast polarity was inversed^30^. However, if this explanation was true, we should have also found reversed effects in switched trials, i. e. longer looking times to the previously cued object. As this was not the case, perceptual weight alone cannot account for the effects found in same position trials.

A second possible influential factor is movement. Farroni, *et al*.^10^ showed that movement is a prerequisite for gaze cues to affect young infants’ attention. In the reversed contrast condition, it seems possible that infants’ attention was guided by the movement of the white pupil which may have led to an enhanced encoding of the cued object. This facilitated object encoding may appear only in same position trials as those trials are much easier to process than switched trials. No additional comparison of the location of the objects became necessary in same position trials. Hence, the effect of movement on infants’ object encoding must have been weaker or less robust than the effect of schematic eyes, as no signs of facilitated object encoding were found in switched trials.

Our results are in line with previous studies showing a specific sensitivity of infants to the black and white contrast of schematic eyes^15, 29^. We expand previous work as we were able to show that the function of such an early sensitivity may be to promote object encoding in infancy. A possible neural basis of this early sensitivity may be the existence of a specified neural system that responds to gaze direction like the suggested DAD^18^ or EDD^7^. The function of such a system consequently is not only to register the focus of attention of another person, but may also be to enhance processing of socially cued objects. Our data suggest that the specific sensitivity of this system is based on low-level features as a simple reversion of polarity disrupts or at least weakens the influence of the cue on infants’ object encoding. The DAD proposed by Perret and colleagues states that, in addition to eye-gaze direction, head and body posture are used to infer the focus of attention of another person^18^. In our study, we focused on the role of eye-gaze contrast polarity. Hence, we cannot draw any conclusion about the role of head and body posture for infants’ object processing. However, there is evidence that the posture of the head influences infants’ object encoding in the same way as eye-gaze direction does^22^.

Using eye tracking, we cannot proof the existence of the proposed neuronal systems in the infant brain. Still, our results highlight the specific sensitivity to eye-gaze cues in early infancy and its influence on object-directed attention. Whether this early sensitivity builds the basis for triadic representations or Theory of Mind as proposed in the Mindreading System by Baron-Cohen^7^ remains unclear. In our study, we did not test such relations. Results of longitudinal studies investigating the relation between early gaze following and later Theory of Mind/intention understanding show mixed results^33–36^. Another possible function of infants’ early sensitivity for eye-gaze cues and the subsequent facilitated encoding of cued objects is to promote social learning processes like imitation in infancy. Social cues like eye gaze may enable the infant to concentrate specifically on the object that is in the focus of attention of another person.

The results of our study challenge accounts which assume that the context of a face is required for effective gaze cueing effects^10^ or enhanced learning processes^3^. It is currently unclear why inverting a face interferes with infants’ gaze processing. In adults it has been suggested that perceiving inverted faces recruits neurons dedicated to processing objects^37, 38^. It is possible that infants process inverted faces rather as objects than as faces and are not able to extract information from the eyes when embedded in the context of an inverted face. It is also possible that the responses of face-sensitive and gaze-sensitive neurons interfere in the context of an inverted face^12^.

We conclude that no facial context is required for infants to use gaze direction as an attentional cue. Our results are in line with the existence of a low-level-information-based gaze processing system like the EDD or DAD to account for human infants’ sensitivity to gaze cues. Other factors like perceptual load or motion may guide infants’ attention but influence object encoding to a weaker extent.

## Electronic supplementary material

Dataset 1

Dataset 2
